# F98. HYPOVITAMINOSIS D IN SCHIZOPHRENIA: ASSOCIATED CARDIOVASCULAR RISK

**DOI:** 10.1093/schbul/sby017.629

**Published:** 2018-04-01

**Authors:** Rahma Nefzi, Amine Larnaout, Hanen Ben Ammar, Emira Khelifa, Amina Aissa, Zouhaier El Hechmi

**Affiliations:** Razi Hospital

## Abstract

**Background:**

Vitamin D modulate the course of many neurologic diseases and conditions. Moreover, the prevalence of vitamin D deficiency might be higher in psychiatric patients, in particular with schizophrenia.

Likewise, there is an inverse relationship between vitamin D levels and several cardiovascular risk factors, including the metabolic syndrome, that patients with schizophrenia are predisposed to develop. It is within this framework that this study aims to explore the relationship between vitamin D levels in a cohort of Tunisian patients with schizophrenia and to determine the cardiovascular risk according to whether they had hypovitaminosis D or not.

**Methods:**

A cross-sectional and retrospective descriptive study was conducted at the “F” psychiatry department at the Razi Hospital, Manouba over a twelve-month period from June 1st, 2015 to May 31st, 2016, including 80 patients with schizophrenia in period of clinical remission. The evaluation focused on anthropometric parameters and cardiovascular risk factors. A dosage of vitamin D was performed.

**Results:**

The patients had an average age of 42.5 years and 70% were male. 25 patients had metabolic syndrome. 49% of patients had vitamin D insufficiency and 51% had vitamin D deficiency. Vitamin D levels had not been affected by the clinical characteristics of the disease. However, there was no significant association between vitamin D levels and metabolic syndrome. A significant negative correlation was found between the total sum of the various cardiovascular risk factors and the vitamin D deficiency (p <0.001).

**Discussion:**

In our study, all patients had vitamin D levels below the recommended levels. 25 patients (31%) met the criteria for metabolic syndrome. All our patients had at least one cardiovascular risk factor. The majority (33% and 27%) had respectively three or four FRCV. 10% had more than five concurrent FRCVs. This result has been described in many studies. Indeed, in patients with schizophrenia, the cardiometabolic risk seems to increase continuously. Several European studies have reported a prevalence of metabolic syndrome ranging from 28% to 37% in patients with schizophrenia. Higher rates of 43% and 46% were reported respectively in the United States and Canada. Moreover, with schizophrenia have an increased risk of sudden death and are 2 to 4 times more likely to die prematurely compared to the general population. These results have been explained with a multicausal model focusing on genetics, lifestyle, smoking, diet and sedentary behavior as well as by the side effects of antipsychotics known to induce weight gain and aggravate symptoms. risk factors for cardiometabolic disease, although studies in naïve patients reflect various abnormalities early on. However, several studies confirm that certain metabolic abnormalities may occur in schizophrenic patients naive to any antipsychotic treatment.

This result is consistent with current literature data that highlight increased metabolic and cardiovascular risk in vitamin D deficiency. Indeed, in the general population, vitamin D deficiency is an important risk factor for cardiometabolic disease. The majority of cohort studies have reported an increase in the incidence of cardiovascular disease in people with low vitamin D levels.

